# Effectiveness of measuring tension during arthroscopic rotator cuff repair

**DOI:** 10.1186/s40634-021-00341-2

**Published:** 2021-03-16

**Authors:** Shin Yokoya, Yoshihiro Nakamura, Yohei Harada, Hiroshi Negi, Ryosuke Matsushita, Norimasa Matsubara, Yasuhiko Sumimoto, Nobuo Adachi

**Affiliations:** Department of Orthopaedic Surgery, Graduate School of Biomedical and Health Sciences, Hiroshima University, Kasumi 1-2-3, Minami-ku, Hiroshima, Japan

**Keywords:** Rotator cuff tear, Arthroscopic rotator cuff repair, Repair tension, Failure rate, Tension meter, Prognostic factor

## Abstract

**Purpose:**

Arthroscopic rotator cuff repair (ARCR) for relatively small rotator cuff tears (RCTs) has shown promising results; however, such surgery for larger tears often results in failure and poor clinical outcomes. One cause of failure is over-tension at the repair site that will be covered with the tendon stump. Reports on the clinical outcomes using ARCR with tension ≤ 30 N are lacking. This study aimed to evaluate ARCR outcomes and failure rates using less tension (30 N) and to assess the prognostic factors for failure.

**Methods:**

Our study group comprised of 118 patients who underwent ARCR for full-thickness RCTs with full tendon stump coverage of the footprint with a tension of ≤ 30 N, measured using a tension meter; no additional procedures, such as margin convergence or footprint medialisation, were performed. The failure rate was calculated, and the prognostic factor for failure was assessed using multivariate regression analyses.

**Results:**

There were seven cases of failure in the study group. Postoperatively, flexion and internal rotation ranges of motion, acromiohumeral interval, muscle strength, and clinical results improved significantly. Using multivariate regression analyses, intraoperative concomitant subscapularis tendon lesion and pre-operative infraspinatus tendon retraction, assessed using radial-sequence magnetic resonance imaging, were significantly correlated with post-ARCR failure using less tension (*p* = 0.030 and *p* = 0.031, respectively).

**Conclusion:**

ARCR is likely to succeed for RCTs that can be extracted using tension ≤ 30 N. However, cases with more severe subscapularis tendon lesions and those with high infraspinatus tendon retraction may show surgical failure.

**Level of evidence:**

LEVEL IV Retrospective case series

## Background

Rotator cuff tears (RCTs), often occurring in older people, are a possible cause of shoulder pain. When first-line conservative therapy is ineffective, arthroscopic rotator cuff repair (ARCR) is widely performed. Excellent results have been reported using ARCR for relatively small RCTs; however, in patients with larger tears, ARCR has frequently resulted in failure and poor clinical outcomes [4, 8, 12, 32]. One cause of failure has been considered to be due to over-tension at the repair site. Davidson and Rivenburgh reported poor clinical results when tears were repaired under a tension of > 8 lb (approximately 35 N) [5]. Therefore, we developed a method of muscle advancement by elevating the supraspinatus and infraspinatus muscles from the scapula and advancing these muscles laterally when the tension at the repair site was > 30 N based on previous studies [5, 18, 25, 36] and reported a lower ARCR failure rate in patients using this advanced procedure [36]. However, it is unknown to what extent clinical results and failure rates could be improved using ARCR with less tension, that is, at ≤ 30 N. Kim et al. reported that the tension on the repair site in one treatment group had resulted in post-ARCR failure when using significantly higher tension than that in a healed group [18]; however, reports concerning the results of cuff integrity and clinical outcomes using ARCR with tension ≤ 30 N are lacking. We aimed to evaluate the outcomes and failure rates of ARCR with tension ≤ 30 N and analyse the prognostic factors causing failure after ARCR under such conditions. The hypothesis was that repairing with a tension of less than 30 N would reduce failure after ARCR, and the larger tears including multiple tendons and the more degenerated RCTs would be the prognostic factors.

## Materials and methods

**Fig. 1:**
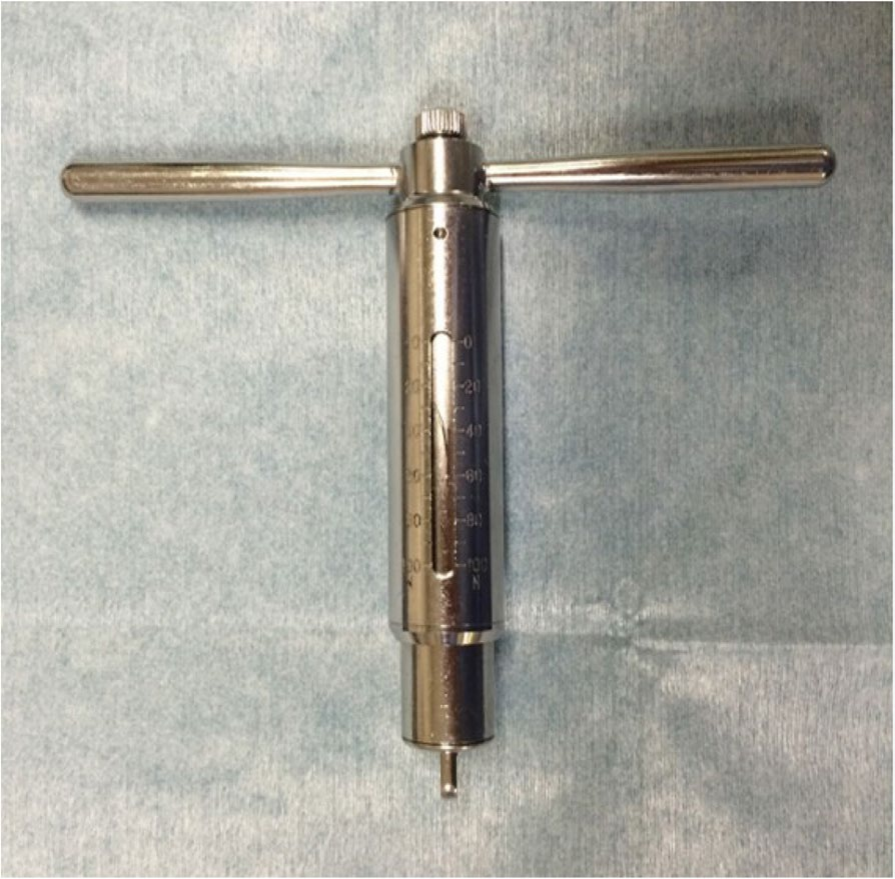
The tension meter (Ligament tensioner 3, MEIRA Corporation, Nagoya, Japan)

These retrospective case seris were approved by our Institutional Review Board (permission number: E-2079). ARCR was performed using the suture bridge technique for 349 consecutive patients diagnosed with RCTs between October 2010 and June 2018, who could be followed up for at least 2 years. Patients with isolated subscapularis (SSC) tendon tears, osteoarthritis or rheumatoid arthritis, and/or partial-thickness RCTs or those requiring surgical revision after a failed repair were excluded from the current study. During this period, no patient had post-operative infection. No medialisation of the footprint [9], margin convergence [2], and interval slide [33] were added for these patients. Furthermore, patients who had undergone ARCR with partial repair [19], muscle advancement [36] or any augmentation, including autograft [24], allograft [10], artificial biomaterial [11, 35, 37, 38], or bone marrow [6, 38] were also excluded. Finally, a total of 118 patients who had undergone ARCR with a bridging suture technique measuring a tension of ≤ 30 N with full coverage of the footprint by the tendon stump, as measured using a tension meter (Ligament tensioner 3, MEIRA Corporation, Nagoya, Japan, Fig. 1), between October 2011 and June 2018 were enrolled in the current study. During this period, when the measured tension exceeded 30 N, we performed ARCR with muscle advancement to extract the retracted tendon [36] and/or with artificial biomaterial (polyglycolic acid sheet [37, 38]) reinforcement to the original footprint without any medialisation to enhance the healing capacity [35]; patients who underwent these procedures were excluded from the present study. In the current study, the average age was 66.1 ± 9.4 (range, 25–82) years. The pre-operative data and several surgical treatments are shown in Table 1.

**Table 1:** Demographic data

	*n* = 118
Sex, n	64 males / 54 females
Age, years	66.1 ± 9.4
Affected arm, n	85 right / 33 left
Follow-up period, months	27.6 ± 10.3
Prevalence of DM	16 ( +): 102 (-)
SSP tendon retraction Boileau Stage I/II/III	25/69/24
ISP tendon retraction Boileau Stage 0/I/II/III	78/20/14/6
Concomitant SSC lesion Lafosse type 0/I/II/III/IV	46/41/27/2/2
Concomitant LHB lesion modified Lafosse Grade I/II/III/IV/V	59/27/12/12/8
Fatty degeneration Goutallier classification Grade 0/1/2/3/4	SSC 65/39/7/6/1 SSP 27/65/23/3/0 ISP 61/45/9/2/1 GFDI 0.76 ± 0.57

The supraspinatus (SSP) and infraspinatus (ISP) tendon retraction grades, assessed preoperatively using radial-sequence magnetic resonance imaging (MRI) as described by Honda et al. [14], were categorised according to Boileau’s classification [31]. The SSC grades and findings were categorised according to the Lafosse classification [20], and the long head of the biceps brachii (LHB) findings was categorised using a modified Lafosse classification [21]. Furthermore, the degree of fatty degeneration (FD) of each rotator cuff muscle was assessed according to the Goutallier classification system [7], and the global fatty degeneration index (GFDI), which was calculated according to the Fuchs report [7].

### Surgical technique

All surgeries were performed by a single surgeon. ARCRs were performed with the patient under general anaesthesia and placed in the beach chair position with the occasional use of an interscalene block. We first examined the glenohumeral joint in the standard manner and performed a synovectomy. If necessary, a capsular release was also performed. When the intraoperative SSC findings were Lafosse type 2 or higher, the arthroscopic repair was performed. When the intraoperative LHB findings were modified Lafosse grade 2 or higher, tenodesis or tenotomy was performed before SSP and/or ISP repair. We then switched to arthroscopic subacromial space observation and performed subacromial decompression and a cuff release to the maximum extent possible.

### Measuring the tension

**Fig. 2:**
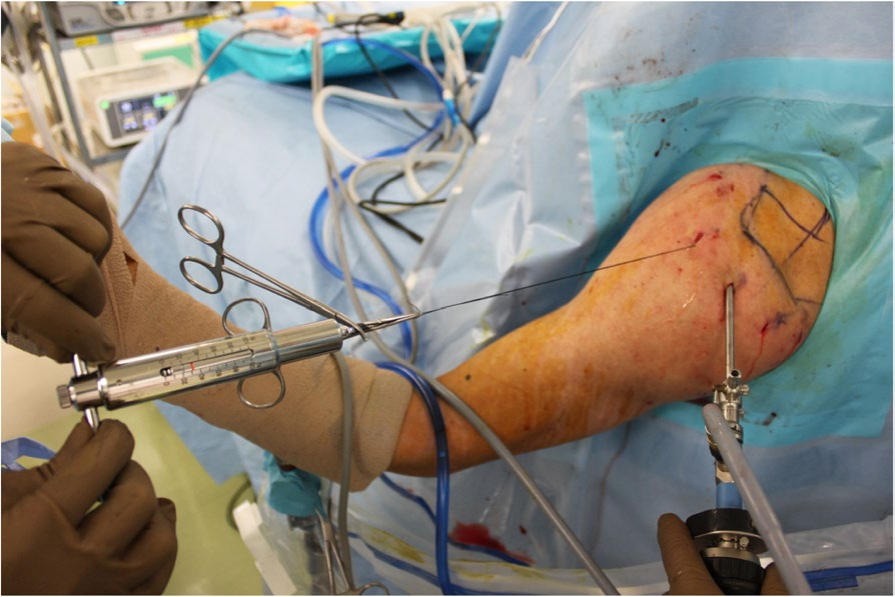
One to three No. 0 nylon threads are applied to the stump of the torn rotator cuff tendon depending on the tear size and are retrieved from the anterolateral portal. Each nylon is pulled laterally by using a tension meter

**Fig. 3:**
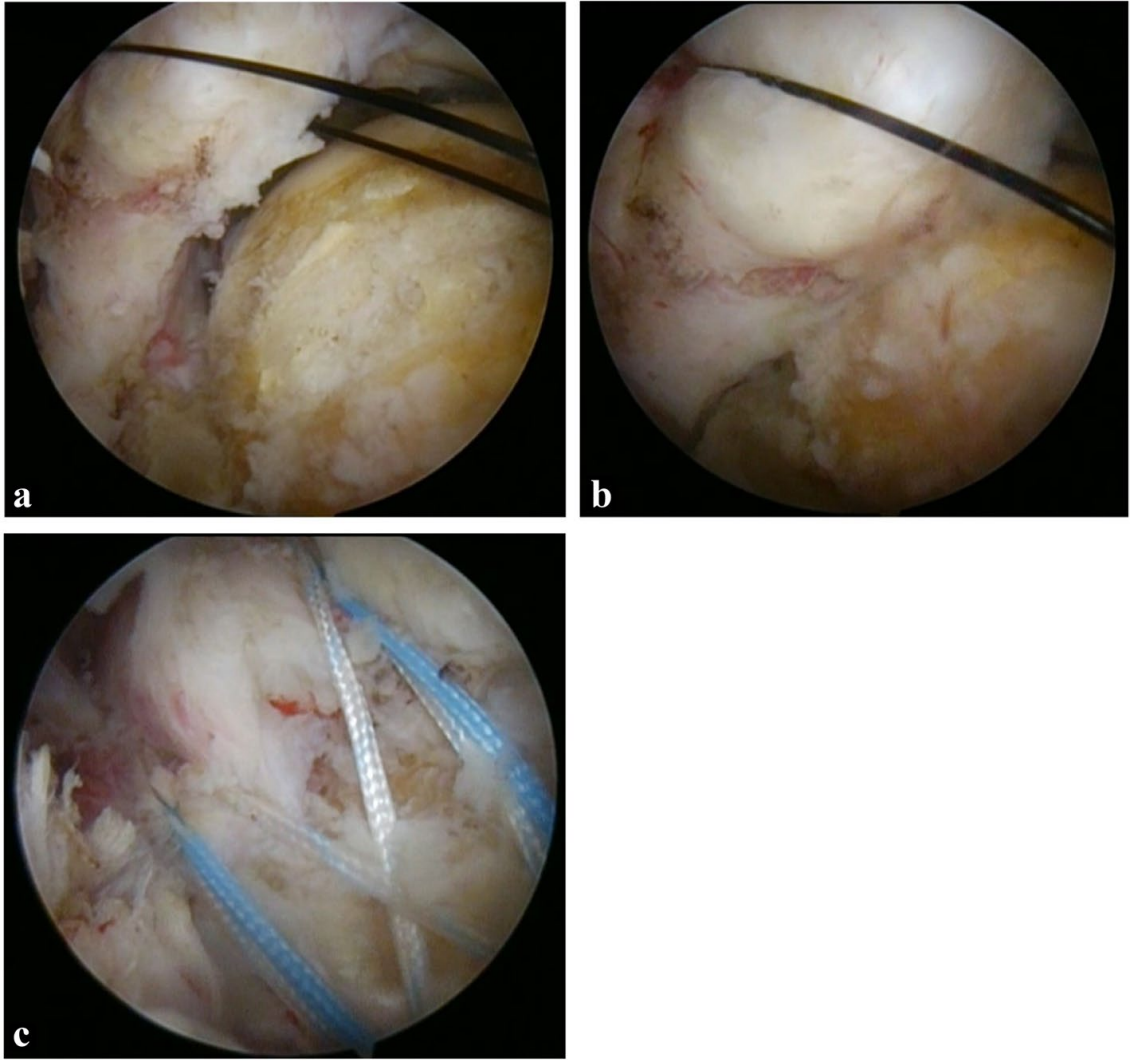
Intraoperative images of the torn cuff. **a** Before pulling the nylon thread. **b** After pulling the nylon thread using a tension of 30 N. **c** After repairing the torn cuff using a suture bridge. When full coverage of the footprint is possible with a tension of ≤ 30 N, arthroscopic rotator cuff repair (ARCR) is performed using the suture bridge technique

Intraoperative tendon excursion has been checked in a 30° abduction position with a muscle relaxant. At first, the types of rupture, such as crescent-, L-, reverse L-, or U-shaped [3], were checked by reducing the torn cuff tendons with a suture retriever. Between one and three No. 0 nylon threads were applied approximately 1 cm from the stump of the torn rotator cuff tendon using a suture hook device, depending on the tear shape or size. Usually, one thread was used for an isolated SSP tear, two threads for a combined SSP and anterior section of the ISP tendon tear, and three threads for full-width tendon tears of the SSP to ISP tendons. The nylon threads were retrieved from the antero-lateral portal, and each nylon thread was pulled laterally (Fig. 2) as the torn cuffs were reduced to the footprint originally using a tension meter. The tension required for the tendon stump to cover the entire footprint was then measured. When full coverage of the footprint cannot be achieved, even when applying a tension of ≥ 30 N, ARCR with muscle advancement was performed [36]. When full coverage of the footprint was possible with a tension ≤ 30 N (Fig. 3a, b), ARCR was performed using a suture bridge technique, which we have previously reported as a modified medial double-pulley technique (Fig. 3c) [36]. When there were two medial anchors, one double pulley was created, and when there were three medial anchors, three double pulleys were created. In such cases, there were always two lateral anchors. The sutures from the suture anchors were penetrated at 2 cm medial to the edge of the torn tendon. If full coverage of the footprint by the tendon stump could be achieved after suture bridge repair completion, the repair was regarded as an anatomical repair. A summary of treatment methods of SSC repair and LHB lesions, with or without capsular release, completion of anatomical repair or not, and bridge configuration pattern is presented in Table 2.

**Table 2:** A summary of the surgical treatment

	*n* = 118
SSC treatment	None:55
	Debridement: 13
	Single repair: 24
	Bridge repair: 22
	PMT: 2
LHB treatment	None: 61
	Tenotomy: 38
	Tenodesis: 12
	Rupture: 7
Capsular release	release ( +): 34
	release (-): 84
Anatomical or non-anatomical repair	anatomical: 92
	non-anatomical: 26
Suture bridge configuration	2 × 2: 108
	3 × 2: 10

### Rehabilitation

For all patients, the affected arm was immobilised for 6 weeks using an abduction brace. The post-operative rehabilitation protocol was as follows: passive range of motion (ROM) exercise commenced from week one postoperatively and assisted ROM within 90º abduction commenced from week four. After abduction brace removal, active ROM > 90º abduction and isometric muscle strength exercises commenced. The cuff muscle strength exercise commenced from week twelve. When tenotomy or tenodesis of the LHB had been performed, elbow joint ROM was prohibited for 3 weeks. In patients engaging in heavy manual work or sports enthusiasts, elbow joint ROM was permitted 6 months postoperatively.

### Evaluation

Post-operative cuff integrity was assessed using MRI, as described by Sugaya et al. [32]. Types IV and V, according to the Sugaya classification, were regarded as surgical failures. The failure rates were then calculated. The current groups were also sub-divided into healed and failed groups to assess the prognostic factors for ARCR failure. These evaluations were conducted by co-authors blinded about the surgical intervention.

Acromiohumeral interval (AHI) values were measured using pre- and post-operative plain radiographic anteroposterior views in standing and external rotation (ER)/internal rotation (IR) neutral positions, according to the procedure described by Ianotti et al. [15] for each patient. Pre-and post-operative ROM measurements were performed in anterior flexion and ER using a goniometer. The IR value was measured based on the highest vertebral level that a patient’s thumb could reach behind their back for each patient. The pre- and post-operative clinical outcomes were evaluated using the Constant score, the University of California, Los Angeles Shoulder Rating Scale (UCLA) score, and the numeric rating score (NRS) for shoulder pain. The term “pre-operative” refers to the period immediately before surgery, and the term “post-operative” refers to the last follow-up session. The pre- and post-operative AHI, ROM values and the clinical scores were compared between the healed and failed group, respectively.

Additionally, isometric muscle strength of the affected arm on abduction and ER was measured pre- and post-operatively using a hand-held dynamometer (Microfet 2; Nihon Medix Co., Ltd., Chiba, Japan). These strength measurements were performed in a seated position, at a 45º abduction angle in the scapular plane for abduction strength, and with the upper arm at the side of the body and the elbow in 90º flexion in an ER/IR neutral position to measure ER strength, as has been previously reported [36]. Each measurement was performed three times, and average values were calculated. The muscle strengths were also compared between the healed and failed groups.

To assess prognostic factors for failure in the study group, age, sex, affected side, prevalence of diabetes mellitus (DM), contracture, SSP and ISP tendon retraction, concomitant SSC and LHB injury, AHI, pre-operative ROM and muscle indexes, FD of each muscle, and GFDI were compared between the healed and failed groups, using univariate and multivariate regression analyses.

### Statistical analyses

All values were expressed as means ± standard deviations. Statistical analyses were performed using the Bell Curve for Excel software (Ver. 3.20; Social Survey Research Information Co., Ltd., Tokyo, Japan). Comparisons between pre- and post-operative values in each group were performed statistically using paired t-test. Comparisons between the healed and failed groups were assessed statistically using Mann–Whitney’s U test. Multivariable logistic regression analysis was used to determine prognostic factors for failure after ARCR focusing on the items that had significant differences in univariate analyses and some items previously reported as prognostic factors. Statistical significance was set at a *p*-value < 0.05.

## Results

We identified 7 (of 118) cases of failure in the current study (failure rate, 5.9%) (Table 3). In a comparison between the pre- and post-operative values in the healed group, AHI improved significantly in the study group, from 9.4 ± 1.9 mm preoperatively to 11.7 ± 2.2 mm, postoperatively. Regarding ROM, flexion and IR improved significantly after surgery (from 125.9 ± 34.0 and Th11.7 ± 2.9 preoperatively to 157.1 ± 12.1 and Th10.0 ± 2.5 postoperatively [*p* < 0.001 and *p* < 0.001, respectively]). However, ER did not improve significantly after surgery (from 56.8 ± 17.1 preoperatively to 57.2 ± 16.0 postoperatively, *p* = 0.647). The Constant scores, UCLA scores and NRS all improved significantly from 48.4 ± 14.9, 14.8 ± 4.3, and 6.6 ± 1.8 preoperatively to 83.3 ± 11.9, 32.6 ± 3.2, and 0.9 ± 1.3 postoperatively (*p* < 0.001, < 0.001, and < 0.001, respectively). On the other hand, in the failed group, AHI (from 9.4 ± 2.1 preoperatively to 11.1 ± 4.1 postoperatively, *p* = 0.098), all ROM (from 139.3 ± 33.2, 56.4 ± 12.8, and Th12.5 ± 2.9 preoperatively to 127.9 ± 35.9, 52.9 ± 12.2, and Th11.7 ± 2.9 postoperatively, *p* = 0.567, 0.376, and 0.486, respectively), Constant score, and UCLA score (from 54.0 ± 18.0, and 17.0 ± 5.9 preoperatively to 65.2 ± 23.3, and 23.1 ± 9.2 postoperatively, *p* = 0.315, and 0.140, respectively) showed no improvement postoperatively, except for the NRS (from 7.0 ± 2.1 preoperatively to 3.1 ± 2.6 postoperatively, *p* = 0.013). In the comparison between the healed and failed groups, postoperative flexion angle, Constant score, UCLA score, and NRS had significant differences (*p* = 0.003, 0.008, 0.002, and 0.005, respectively) in spite of no significant differences in all preoperative values (*p* = 0.860, 0.193, 0.755, 0.648, 0.288, 0.342, and 0.663, respectively)and postoperative AHI, ROM of ER, and IR (*p* = 0.913, 0.313, and 0.846, respectively). The results of the AHI and ROM and clinical scores are summarised in Table 4.

**Table 3:** Cuff integrity and failure rate

Sugaya classification	*n* = 118	
Type I	49	111
Type II	54	
Type III	8	
Type IV	1	7
Type V	6	
Failure rate	5.9%

**Table 4:** Results of the AHI, ROM and clinical scores

	Healed group (*n* = 111)	Failed group (*n* = 7)	*p* value
Pre AHI, mm	9.4 ± 1.9	9.4 ± 2.1	0.860
Post AHI, mm	11.7 ± 2.2	11.1 ± 4.1	0.913
*p* value	< 0.001*	0.098	
Pre flexion, °	125.9 ± 34.0	139.3 ± 33.2	0.193
Post flexion, °	157.1 ± 12.1	127.9 ± 35.9	0.003†
*p* value	< 0.001*	0.567	
Pre ER,°	56.8 ± 17.1	56.4 ± 12.8	0.755
Post ER, °	57.2 ± 16.0	52.9 ± 12.2	0.313
*p* value	0.647	0.376	
Pre IR, spine level	Th11.7 ± 2.9	Th12.5 ± 2.9	0.648
Post IR, spine level	Th10.0 ± 2.5	Th11.7 ± 2.9	0.846
*p* value	< 0.001*	0.486	
Pre constant score	48.4 ± 14.9	54.0 ± 18.0	0.288
Post constant score	83.3 ± 11.9	65.2 ± 23.3	0.008†
*p* value	< 0.001*	0.315	
Pre UCLA score	14.8 ± 4.3	17.0 ± 5.9	0.342
Post UCLA score	32.6 ± 3.2	23.1 ± 9.2	0.002†
*p* value	< 0.001*	0.140	
Pre NRS	6.6 ± 1.8	7.0 ± 2.1	0.663
Post NRS	0.9 ± 1.3	3.1 ± 2.6	0.005†
*p* value	< 0.001*	0.013*	

^*^Statistically significant between the pre- and post-operative values (*p* < 0.05)

^†^Statistically significant between the healed and failed groups (*p* < 0.05)

The abduction and ER muscle strengths in the healed group improved significantly (from 33.1 ± 17.7 and 43.0 ± 18.2 preoperatively to 68.7 ± 26.6 and 65.9 ± 22.5 postoperatively, *p* < 0.001, < 0.001, respectively), despite no significant differences in the failed group (from 36.9 ± 21.7 and 37.1 ± 21.5 preoperatively to 38.6 ± 35.5 and 54.1 ± 32.4 postoperatively, *p* = 0.872, 0.127, respectively). IR muscle strengths in the healed group also improved significantly (from 77.8 ± 31.3 preoperatively to 113.5 ± 37.1 postoperatively, *p* < 0.001) despite no significant difference in the failed group (from 71.0 ± 44.4 preoperatively to 91.1 ± 44.1 postoperatively, *p* = 0.090) Comparison between the healed and failed groups showed no significant differences in all strengths (pre abduction *p* = 0.405, pre ER *p* = 0.547, pre IR *p* = 0.392, post ER *p* = 0.144, and post IR *p* = 0.158, respectively) except for post-operative abduction strength (*p* = 0.025). The results of the muscle strength indexes are summarised in Table 5.

**Table 5:** Results of isometric muscle strength concerning each group

	Healed group (*n* = 111)	Failed group (*n* = 7)	*P*-value
Pre AbdS, N	33.1 ± 17.7	36.9 ± 21.7	0.405
Post AbdS, N	68.7 ± 26.6	38.6 ± 35.5	0.025†
*p* value	< 0.001*	0.872	
Pre ERS, N	43.0 ± 18.2	37.1 ± 21.5	0.547
Post ERS, N	65.9 ± 22.5	54.1 ± 32.4	0.144
*p* value	< 0.001*	0.127	
Pre IRS, N	77.8 ± 31.1	71.0 ± 44.4	0.392
Post IRS, N	113.5 ± 37.1	91.1 ± 44.1	0.158
*p* value	< 0.001*	0.090	

*Abds* abduction strength, *ERS* external rotation strength, *IRS* internal rotation strength

^*^Statistically significant between the pre- and post-operative values (*p* < 0.05)

^†^Statistically significant between the healed and failed groups ( *p* < 0.05)

The results of univariate comparisons between the healed and failed groups in relation to pre-operative assessments in the study group are shown in Table 6. Only ISP tendon retraction, assessed using radial-sequence MRI, and intraoperative concomitant SSC lesion evaluated by Lafosse classification showed significant differences (*p* = 0.012, and 0.006, respectively). Multivariate regression analysis results also indicated a significant correlation between the pre-operative ISP tendon retraction and concomitant SSC lesion (*p* = 0.031 and *p* = 0.030, respectively). The odds ratios and 95% confidence intervals for these variables were 19.4 and 1.31–287.3 for ISP tendon retraction, and 4.41 and 1.16–16.8 for the concomitant SSC lesion respectively (Table 7).

**Table 6:** Results of univariate analyses between the healed and failed groups

	Healed group (*n* = 111)	Failed group (*n* = 7)	*P*-value
Age, years	66.1 ± 9.2	65.6 ± 12.9	.673
Sex, male: female	59: 52	5: 2	.581
Affected side, right: left	80: 31	5:2	.967
Prevalence of DM	15 ( +): 96 (-)	1 ( +): 6 (-)	.952
SSP retraction, Boileau I/II/III	24/65/22	1/4/2	.993
ISP retraction, Boileau 0/I/II/III	77/16/14/4	1/4/0/2	.012*
Concomitant SSC lesion Lafosse type 0/I/II/III/IV	44/39/26/1/1	2/2/1/1/1	.006*
Concomitant LHB lesion modified Lafosse I/II/III/IV/V	56/25/12/12/6	3/2/0/0/2	.648
Pre AHI, mm	9.41 ± 1.85	9.35 ± 2.09	.862
Pre flex, deg	125.9 ± 34.0	139.3 ± 33.2	.194
Pre ER,deg	56.8 ± 17.1	56.4 ± 12.8	.758
Pre IR, spine level	Th11.7 ± 2.9	Th12.5 ± 2.9	.348
Pre FD of SSC	61:38:7:5:0	4:1:0:0:0	.970
Pre FD of SSP	25:63:20:3:0	2:2:3:0:0	.683
Pre FD of ISP	58:44:8:1:0	3:1:1:1:1	.252
Pre GFDI	0.73 ± 0.51	1.24 ± 1.13	.154
Pre constant score	48.4 ± 14.8	54.0 ± 18.0	.502
Pre UCLA score	14.8 ± 4.3	17.0 ± 5.9	.360
Pre NRS	6.6 ± 1.8	6.8 ± 2.2	.914

^*^Statistically significant between the healed and failed groups (*p* < 0.05)

**Table 7:** Results of multivariate regression analyses

	Partial regression coefficient	Odds ratio	95% CI	*P*-values
Age	0.22	1.24	0.95–1.64	.120
ISP retraction	2.97	19.4	1.31–287.3	.031*
Concomitant SSC lesion	1.48	4.41	1.16–16.8	.030*
Pre constant score	0.21	1.24	0.99–1.55	.064
Pre NRS	0.83	2.29	0.72–7.31	.160

^*^Statistically significant between the healed and failed groups (*p* < 0.05)

## Discussion

This study investigated clinical outcomes and ARCR failure rates and assessed prognostic factors to determine post-ARCR failure using tension ≤ 30 N to restore the original footprint without any additional treatment, such as footprint medialisation or muscle advancement. Our findings showed that this procedure achieved good clinical outcomes when the repaired tendon completely healed. Flexion angle and clinical scores significantly improved in the healed group than in the failed group. Even after such surgeries, the prognostic factors for failure were the concomitant SSC lesion and ISP tendon retraction before surgery.

Many studies have reported high failure rates of ARCR for large RCTs [4, 8, 12, 32]. Until 2011, we performed ARCR with a thorough cuff release, footprint medialization [9], margin convergence [2], or autologous patch graft using fascia lata [24] for large RCTs. However, we frequently encountered failure after ARCR for these large RCTs, and a review of our surgical methods was considered necessary. A decision was made to repair the torn cuffs at a tension of ≤ 30 N, in response to Davidson and Rivenburgh’s study reporting high-tension repairs > 8 lb [5] (35.6 N) were associated with poor outcomes. Although some studies have reported using a surgical technique in which over-tension (≥ 30 N) was not applied to avoid failure [25, 36], no study has investigated the failure rate for ARCR in completely repaired cuffs using a tension of ≤ 30 N. Kim et al. measured the tensions needed to repair the cuff of the original footprint and reported a failure rate of 18.2% after ARCR with single-row repair. They found a significant negative correlation between repair tension and cuff healing [18]. The tension was significantly lesser in the healed group than in the failed group (26.5 ± 22.9 N vs. 37.2 ± 22.1 N, *p* = 0.04). This report suggested that repairs with tensions ≤ 30 N could be successfully undertaken. Of the 118 patients in our study, 24 (approximately 20%) with a stage III Boileau classification (considered to be large tears) were included. A failure rate of 5.9% can be considered a relatively good result compared with those reported by previous studies [23, 26, 28, 29, 34].

Generally, several factors have been reported as causes of failure after ARCR, such as age [1, 34], tear size [4, 8, 12, 32], tendon quality [17], or muscle FD [16, 29]. Using univariate analysis, Neyton et al. reported that a history of cigarette smoking was a risk factor for failure [26]. Ishitani et al. introduced a novel classification for the tendon stump, graded as the signal intensity compared with that of the deltoid, and reported that type 3 stumps (tendon signal intensity was higher than that of the deltoid) had significantly higher failure rates, using logistic regression analysis [17]. Scheiderer et al. reported a higher critical shoulder angle of > 38° was a risk factor for failure [28]. We conducted a multivariate regression analysis of the risk factors for cases with failure despite ARCR with tension ≤ 30 N and found that concomitant SSC lesion and ISP tendon retraction were prognostic factors for failure.

Oh et al. reported that FD of the ISP muscle was the most independent prognostic factor affecting the anatomical outcome [27]. Shimokobe et al. reported that the active ER angle for postero-superior RCTs was a risk factor, using multivariate logistic regression analysis [30]. Their results showed that the loss of the active ER angle correlated with the FD of the ISP muscle. Therefore, the ISP muscle is likely to be associated with post-ARCR failure. In our study, the FD of the ISP muscle was not a prognostic factor for failure, whereas ISP tendon retraction was. To date, ISP tendon retraction as a risk factor has not been reported because it has been challenging to evaluate the ISP tendon retraction degree using conventional MRI. Honda et al. stated that radial-sequence MRI was particularly useful in assessing postero-superior RCTs [14]. Using radial-sequence MRI to evaluate the RCTs, we identified ISP tendon retraction as a prognostic factor.

Few studies have reported concomitant SSC lesion as a risk factor for failure; however, Herring et al. reported that the number of ruptured tendons correlated with failure, with an odds ratio of 5.83 for three-tendon tears [13]. We consider that even a repaired tendon whose quality and excursion could be considered satisfactory is likely to fail when the concomitant SSC condition is worse because the SSC’s superior aspect and SSP’s anterior aspect are usually continuous [22].

Whilst our study had certain strengths, it also had some limitations. First, this was a retrospective and non-randomised study; therefore, selection bias was likely. Second, the tension on the repair must be influenced by the type and correct reposition of the cuff tears and the tendon quality. Although ARCRs were consistently performed in a same arm position under the same anaesthesia, the type of tendons was different in each case, and non-anatomical repairs remaining uncovered footprint were achieved in some cases. In addition, the tendon qualities were not evaluated intraoperatively. These factors may also have influenced the results. Furthermore, the suture bridge repair itself may lead to over-tensioning of the construct compared to single-row repairs. The problem, however, was minimized by uniting all repair techniques to the suture bridge techniques. Third, we performed cuff releases to the maximum extent possible for all cases. However, it is not possible to prove that the standardisation was successful before repair. Finally, Kim et al. reported no validated method for measuring the repair tension after repair [18]. Therefore, it is unclear whether the intraoperatively measured values in this study accurately reflect the repair tension. This time, the tension required for complete coverage of the footprint with cuff stump before the repair was regarded as repair tension during the cuff repair, similar to the previous reports [5, 18, 25, 35, 36].

## Conclusions

ARCR with suture bridge technique is likely to succeed (failure rate: 5.9%) for RCTs that can be extracted using tension ≤ 30 N before repair. The AHI, ROM except for ER, all functional scores, and isometric muscle strengths improved significantly after complete ARCR. The multivariate regression analysis results indicated that the concomitant SSC lesion and pre-operative ISP tendon retraction were prognostic factors for failure even in ARCR with a tension of ≤ 30 N.

## Abbreviations

RCTs: Rotator cuff tears
ARCR: Arthroscopic rotator cuff repair
SSC: Subscapularis
SSP: Supraspinatus
ISP: Infraspinatus
MRI: Magnetic resonance imaging
LHB: Long head of the biceps brachii
FD: Fatty degeneration
GFDI: Global fatty degeneration index
ROM: Range of motion
AHI: Acromiohumeral interval
ER: External rotation
IR: Internal rotation
UCLA: University of California, Los Angeles
NRS: Numeric rating score
DM: Diabetes mellitus
